# Cytogenetics to functional genomics: six decades journey of Professor P.K. Gupta

**DOI:** 10.1111/pbi.13795

**Published:** 2022-03-22

**Authors:** Pawan L. Kulwal, Reyazul Rouf Mir, Rajeev K. Varshney

**Affiliations:** ^1^ State Level Biotechnology Centre Mahatma Phule Krishi Vidyapeeth (Agricultural University) Rahuri India; ^2^ Division of Genetics & Plant Breeding Faculty of Agriculture Sher‐e‐Kashmir University of Agricultural Science and Technology, Kashmir Sopore India; ^3^ 5673 Centre of Excellence in Genomics and Systems Biology International Crops Research Institute for the Semi‐Arid Tropics Patancheru India; ^4^ 5673 State Agricultural Biotechnology Centre Centre for Crop & Food Innovation Food Futures Institute Murdoch University Murdoch WA Australia

**Keywords:** cytotaxonomy, cytogenetics, mutation breeding, genetic resources, markers, SSRs, SNPs, genetic maps, QTLs, GWAS, marker‐assisted selection, gene pyramiding

## Abstract

We had the fortune of starting our scientific/research careers in the Molecular Biology and Crop Biotechnology Laboratory of Professor P.K. Gupta at Ch. Charan Singh University, Meerut, UP, India. Here, we describe the most important scientific contributions of our beloved mentor in the area of cytotaxonomy, cytogenetics, mutation breeding, quantitative genetics, molecular biology, crop biotechnology and plant genomics, on his 85th birthday. Important contributions made in the development and use of genomics resources including the development and use of different kinds of molecular markers, genetic and physical mapping, quantitative trait locus (QTL) interval mapping, genome‐wide association mapping and molecular breeding including marker‐assisted selection have been briefly summarized. Efforts have been also made to give readers a glimpse of important contributions in the study of cytology/apomixis of grasses, cytotaxonomic studies in asteraceae/fabaceae, nuclear/repetitive DNA content in *Lolium*, interspecific/intergeneric relationships involving the genus *Hordeum* and re‐examining taxonomy of the tribe Triticeae.

## Introduction

**Figure 1:**
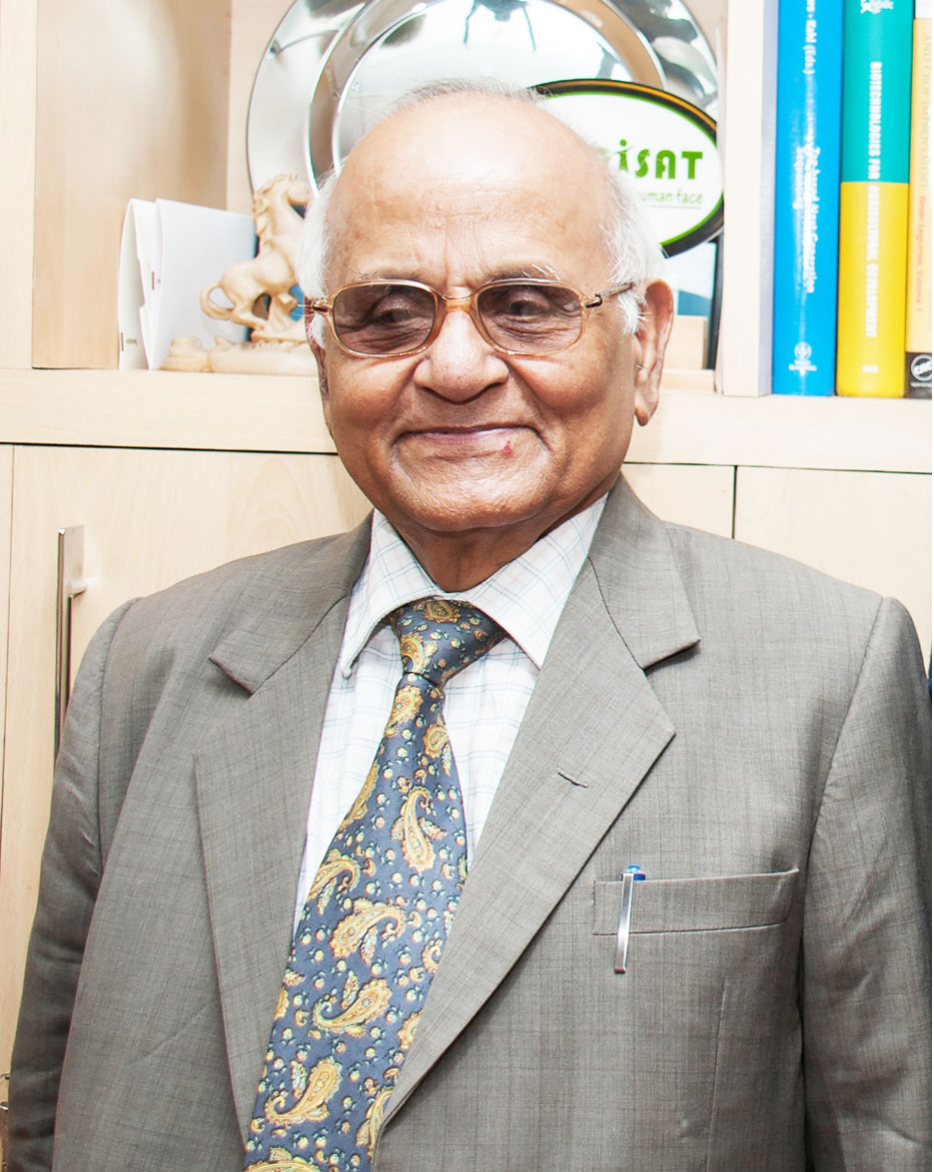
Stand‐alone picture of Professor P.K. Gupta.

Often a scientist, starting his/her career in one field of specialization, completes his/her career in the same field of research. Professor Gupta (Box 1, Figure 1) is an exception to this general trend and continued modifying his research interest in the upcoming/emerging, but related areas of plant biology/genetics without losing his major focus in genetics and plant breeding. Over the years, he worked in several related areas including cytogenetics, genetics, mutation breeding, quantitative genetics, molecular biology, crop biotechnology and genomics (Table 1). For achieving this, he also successfully completed a number of research projects.

Box 1 **Life sketch**

Professor Pushpendra Kumar Gupta (Figure 1) was born on December 14, 1936 in the city of Saharanpur, Uttar Pradesh (U.P.), India and completed his school education up to grade 12 from the same place. Later, he completed his education for his undergraduate (B.Sc.) and postgraduate (M.Sc.) degrees from Agra University, India in 1956 and 1958, respectively. Immediately after his post‐graduation, he joined Agra University and after a brief stint as Lecturer at Meerut College, Meerut and DAV College, Muzaffarnagar, he moved to Gorakhpur University in 1960 where he worked for nine years (1960–69). During this period, largely involving teaching and research in the area of genetics and cytogenetics, he also availed Commonwealth Scholarship for his Ph.D. program, which he completed at the University of Manitoba, Canada in 1967. Although he hardly had any mentor in formal sense, he gives credit for his success to Professor KS Bhargava (Gorakhpur) and Professor RP Roy (Patna) who gave a much desired direction to his career and his entry into the subject of Genetics and Cytogenetics. During his Ph.D. period, he had a good interaction with Professor BC Jenkins (General Secretary, First International Wheat Genetics Symposium that was held at University of Manitoba in 1958) who suggested him the Ph.D. research topic on wheat‐rye substitutions. In 1969, he joined Meerut University, Meerut (now Ch. Charan Singh University, Meerut) initially as Reader in the newly created Division of Plant Sciences and later in 1976 as Professor in the Department of Agricultural Botany (now Department of Genetics and Plant Breeding) with additional responsibility as Dean, Faculty of Agriculture. He superannuated from that position in 1996, and thereafter continued working in the department as an Emeritus Professor.

**Figure 2:**
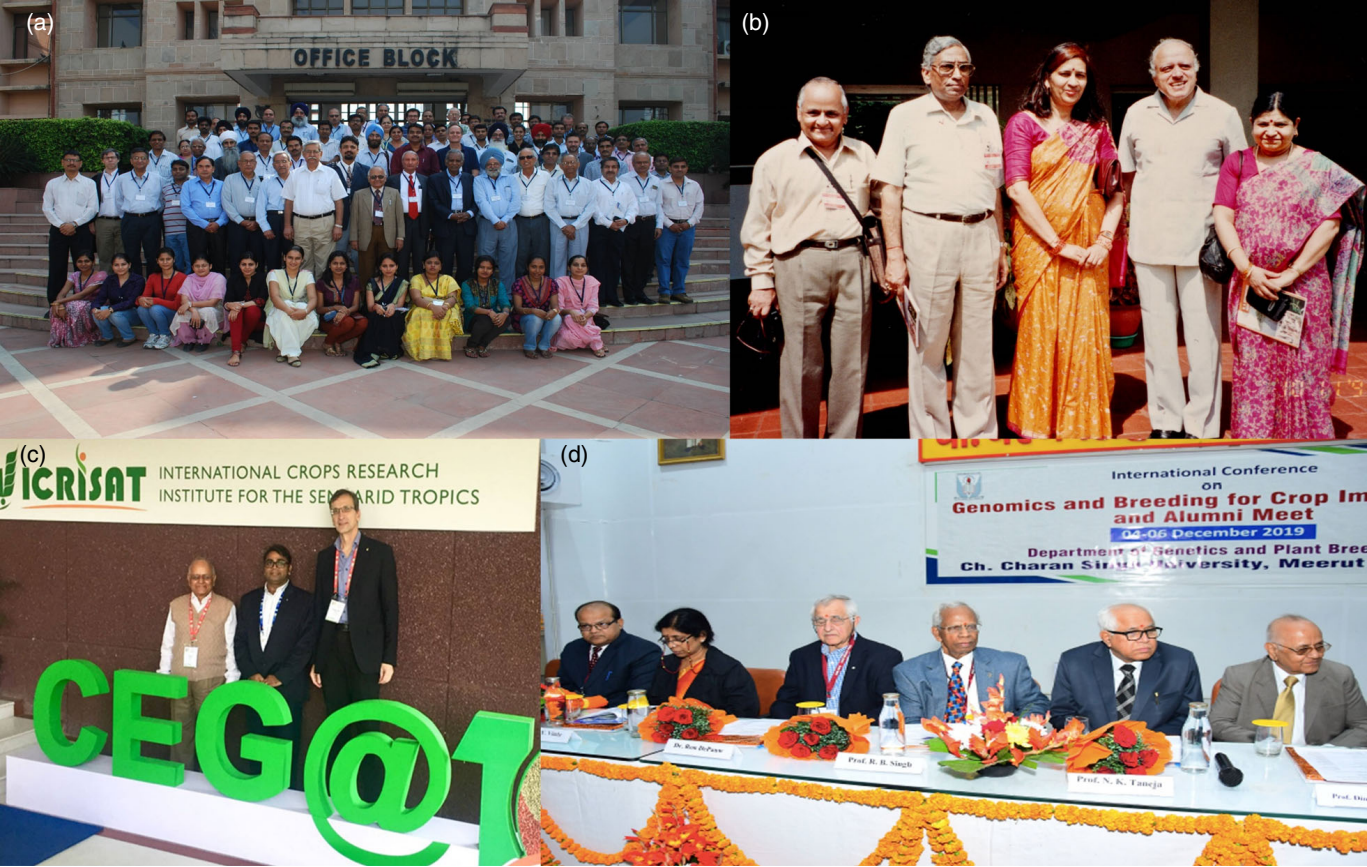
Professor PK Gupta in different national and international conferences. (a) Professor Gupta with several of his colleagues and scientists from India and abroad during the International Conference on Development and Use of Molecular Markers for Crop Improvement organized by his department in New Delhi in October 2011. (b) Professor PK Gupta and his wife Sudha Gupta (extreme right) with Hari Om Agarwal (2nd from left) and his wife (3rd from left) and MS Swaminathan (4th from left) at MS Swaminathan Research Foundation, during a meeting of Indian Academy of Sciences, held in Chennai. (c) Professor PK Gupta along with Prof. Rajeev Varshney (in the middle) and Prof. Andreas Graner (extreme right) during an international conference on “Plant Genomics: Present & Future” at Hyderabad in 2017. (d) Professor Gupta while organising International Conference at Meerut in 2019.

Although, generally it is not easy for a scientist to shift to new and emerging research areas, Professor Gupta, being a voracious reader/writer, could do this without any problem. This was also possible because he was associated with a number of international academic bodies and subscribed to several international journals. This also resulted in an ever‐growing personal library, which became a rich resource for his students, since the university subscribed to a limited number of journals. Since he kept himself abreast with the recent developments in his subject area, he was regularly invited to many national and international seminars and symposia to deliver the invited lecture, which he still continues to do (Figure 2). Therefore, in India, he is also popularly described as an ‘Encyclopedia’ of knowledge in his ever‐changing subject area of genetics and molecular biology. Over six decades of his research journey in the major area of genetics and plant breeding, he touched upon many streams and carried out excellent work (Table 1). Not only he always carried out research in an emerging and ever‐growing broad area of genetics but also raised his department to the international level in the area of wheat genetics and genomics. In doing this, he provided training to several generations of scientists. On his 85th birthday, we present his contributions in diverse but related areas in this article.

**Table 1:** Contribution of Professor P. K. Gupta in different research areas

S. No	Research area	Active years	Aspects
1.	Cytogenetics, genetics	1963–1998	Chromosome studies in Paniceae, *Heteropogon*, rye, wheat, *Cynodon dactylon*, *Dichanthium*, *Carthamus*, *triticale*, *Setaria*, okra, *Digitaria*, *Crotalaria*, *Trigonella*, *Medicago*, barley, etc.; self‐incompatibility, ploidy studies in different species; genetic studies for various traits in potato; brassica, mungbean, lentil, chickpea, pearl millet and wheat
2.	Mutation breeding	1963–1998	Induced mutations in wheat, rye, foxtail millet, *Crotalaria*, pea, sunn hemp, black gram, lentils, etc.
3.	Quantitative genetics, plant genomics and crop biotechnology	1995 onwards	Development and use of molecular markers (SSR, EST‐SSR, STS, AFLP, SAMPL and SNPs) in wheat, SSRs in barley and jute; linkage mapping, QTL IM, GWAS for various traits in wheat and jute; genetic and physical mapping of SSRs in wheat
4.	Bioinformatics	2002 onwards	*In silico* analysis of EST data, homology search, DNA sequencing; identification of novel genes in wheat
5.	Applied genomics	2005 onwards	Marker‐assisted selection for grain quality traits and rust resistance in wheat
6.	Structural and functional genomics	2010 onwards	Transcriptome analysis in wheat, identification of transcription factors
7.	Non‐coding RNA	2013 onwards	miRNAs and lncRNAs in wheat
8.	Epigenetics	2015 onwards	DNA methylation, histone modifications

## Research leadership

### Building the Department of Genetics and Plant Breeding

The Department of Genetics and Plant Breeding (formerly Department of Agricultural Botany prior to 2004) at Ch. Charan Singh University, Meerut (India) was established in 1969, as a part of the Division of Plant Sciences (comprising the subjects of botany, agricultural botany, agronomy and horticulture) of the so‐called Institute of Advanced Studies. In 1973, the Division of Plant Sciences was bifurcated, thus creating separate Departments of Botany and Agricultural Sciences (with agricultural botany, agronomy and horticulture, each with a single faculty member). In this set‐up, Professor Gupta became a part of the Botany Department, but he moved to the Department of Agricultural Sciences as Professor and Head of the Department in 1976. Since he was the sole faculty in the subject of genetics and plant breeding, he made major efforts for strengthening the department on a war footing. This was not easy and involved the development and submission of proposals to the University Grants Commission (UGC) and the State Government of Uttar Pradesh (UP), followed by travelling to Delhi (the national capital) and Lucknow (the state capital) at his own expense to convince the policymakers about the need and desirability of strengthening the department through the sanction of teaching and non‐teaching staff. This resulted in the sanction of additional teaching faculty (two readers and three lecturers), and non‐teaching staff along with important equipment including a ‘Vickers Integrating Microdensitometer’ for DNA estimations, which Meerut University was the first to procure in India. This was the beginning of a journey extending over four decades of active teaching and research in the field of cytogenetics, molecular biology, genetics and plant breeding, thus bringing the department on the world map.

Later, due to his efforts, even after his superannuation, the department was also sanctioned grants by the Department of Science and Technology (DST) under their FIST (‘Fund for Improvement of S&T Infrastructure) program, by UGC under their ‘Special Assistance Programme and by Department of Biotechnology (DBT), Govt. of India in the form of Bioinformatics Infrastructure Facility (BIF).

Due to recognition of the department at the national and international levels, the department could also secure funding for a large number of research projects from various funding agencies including DST, DBT, Government of India, Council for Scientific and Industrial Research, Indian Council of Agricultural Research (ICAR), Department of Atomic Energy, UGC as well as UP State Council of Science and Technology as well as different science academies in India, and National Science Foundation, USA.

During his tenure as Professor of Agricultural Botany and Dean, Faculty of Agriculture, for two decades (1976–1996), significant research was carried out by his group in several areas of research including cytogenetics, mutation breeding and quantitative genetics. During this period, he also wrote several textbooks and also edited some volumes in the area of cytology, cytogenetics, genetics and biotechnology, which are widely read even today. This was possible despite his preoccupation with a variety of administrative activities of the university. However, the most productive phase of his research career started just before his superannuation, when Dr. CR Bhatia, the then Secretary, DBT, Govt. of India invited and sanctioned him adequate funds to establish a Wheat Biotechnology Laboratory (as a part of a multi‐institutional research project on wheat biotechnology). Professor Gupta accepted this challenge and established a Wheat Biotechnology Laboratory, which later became known as Molecular Biology Laboratory (MBL).

Professor Gupta superannuated in 1996, and then worked whole time for research. This activity never slowed down, so he still continues to work full‐time for research, mentoring a number of research students (with some minor teaching load). Consequently, excellent facilities became available for undertaking research work in the field of plant genetics and genomics, crop biotechnology and molecular biology. Later, with the establishment of DBT‐funded BIF, several research projects involving both *in silico* research (in BIF) and wet‐lab work (in MBL) were also successfully completed leading to the publication of a series of papers.

The work on the development and use of molecular markers for wheat breeding, which he initiated as a part of a DBT funded multi‐institutional project in 1996 brought recognitions to the department, which thus became known as an important centre for development and use of molecular markers, genetic dissection of traits and marker‐assisted breeding, primarily in wheat, and with a secondary interest in jute. The research in jute was conducted in collaboration with scientists at ICAR‐Central Research Institute for Jute and Allied Fibres (ICAR‐CRIJAF).

## Research contributions

### Cytotaxonomy and cytogenetics

Often cytogenetics is assumed to include all kinds of studies involving even just the study of chromosomes, but we like to make a distinction between cytotaxonomic and cytogenetic studies. While cytotaxonomy involves the study of chromosome numbers along with chromosome structure and behaviour, cytogenetics deals with the study of heredity through cytology and genetics involving the study of the various aspects of chromosomes and the variations related to their transmission, recombination and expression of the genes carried by them. In his textbook on cytogenetics, Professor Gupta defined cytogenetics as a correlated study of chromosomes and the genes carried by them, so that an important area of cytogenetics is genetic and physical mapping of genes on the chromosome (sometimes utilizing structural and numerical alteration in chromosomes) and transfer of alien genetic variation to cultivated species. Professor Gupta initially worked in the area of cytotaxonomy (sometimes with chemotaxonomy), but later conducted research, which was truly cytogenetics in nature.

#### Cytology of grasses of Gorakhpur

While working as a lecturer at Gorakhpur University, Professor Gupta started his career involving teaching genetics along with preliminary research involving the study of meiosis in local grasses of Gorakhpur, with late Professor RP Roy of Patna University as his mentor; this necessitated his regular visits to Patna University in all his holidays during 1961–1964, before he left for Canada to avail the Commonwealth Scholarship. During these early years of his career, he collected grasses of Gorakhpur, studied meiosis in these grasses, and determined the gametic chromosome numbers leading to his first publication on chromosome numbers in eight species of the tribe Paniceae (Gupta, 1963).

#### Apomixis in some grasses

During his studies of meiosis in grasses, Professor Gupta also observed the occurrence of meiotic irregularities (involving the formation of multivalents) in some grasses from the genera *Dichanthium*, *Bothriochloa* and *Heteropogon*. Further studies revealed that a number of these species (including *Dichanthium annulatum* and *Bothriochloa pertusa*) were facultative apomicts except *Heteropohgon controtu*s, which was an obligate apomict. An offshoot of these species was also the discovery of seasonal variation in the extent of apospory (in terms of the number of aposporic embryo sacs) in *D. annulatum*. These studies were also continued at Meerut during early 1970 when apomixis was studied in *Cenchrus ciliaris*. Another offshoot of these studies included the identification of ecotypic differentiation in *Eleusine indica*, and triploidy in the local doob grass, *Cynodon dactylon*.

Later after returning from Canada in 1967, during the late 1960s and early 1970s Professor Gupta and his group continued the study of meiosis and mitosis (including karyotypes) in a number of members from the families Poaceae, Fabaceae and Asteraceae. Later he picked up a few genera and conducted detailed studies of several genera. The main focus of these studies was to elucidate evolutionary trends based on a study of interspecific and intraspecific variability in these genera/species.

#### Cytotaxonomic studies in Asteraceae and Fabaceae

Professor Gupta also undertook an exhaustive study on cytological aspects of a large number of taxa of the family Asteraceae, with almost one‐third species studied for the first time. He made several interesting observations including the case of *Blumea lacera* where all the 11 chromosomes were observed to be involved in the formation of multivalent, suggesting complex translocation heterozygosity. Subsequently, meiotic studies were continued in a large number of species in several genera, which also included reports of B‐chromosomes in several species of the genus *Crotalaria*.

Professor Gupta also performed cytological studies on the genus *Digitaria* with 325 species distributed all over the world with differences in chromosome numbers. Initially, he performed meiotic studies in four different species of *Digitaria* of which cytogenetic analysis of two species was carried out for the first time. In another species, a new chromosome number was recorded. Two cytotypes of *Digitaria glauca* showed abnormal meiosis (2*n* = 45; 2*n* = 39).

Professor Gupta and his group conducted extensive studies on the cytogenetics of legumes also. In some species, new chromosome numbers were reported and consequently new basic chromosome numbers were proposed. Also, interesting cases of dysploidy were reported in some species. For instance, chromosomes were studied in 28 species of the genus *Indigofera*, 18 species of *Medicago* and 7 species of *Trigonella* of the family Fabaceae.

The cytological and cytogenetic aspects were also studied in the following genera/species of the family Fabaceae: *Cicer arietinum* (chickpea) and its related wild species, *Lens culinaris* (lentil), *Cajanus cajan* (pigeonpea), *Vigna mungo* (black gram) and *Pisum sativum* (garden pea). Standard karyotypes of the above species were prepared for the first time (Gupta and Sharma, 1991). Pachytene karyotypes were also prepared in lentil and *Cicer bijugum*, a wild species related to chickpea. Induced translocations (including the development of a translocation tester set and aneuploids were also analysed in lentils. Interchange trisomics were reported in garden pea.

Artificial induction of polyploidy was another area of his study, which included the development of induced polyploidy in chickpea, lentils, several species of the genus *Crotalaria* and an ornamental, *Zinnia elegans*.

#### Interspecific and intergeneric relationships involving the genus *Hordeum*

During 1984–1987, Professor Gupta spent 3 months every summer working with his old classmate Professor George Fedak of Agriculture Canada in Ottawa, Canada, on a fellowship offered by Canadian International Development Agency (CIDA). Based on the data on meiosis in hexaploid and tetraploid species of *Hordeum*, their respective polyhaploids, and intergeneric–interspecific hybrids, it was inferred that a meiotic pairing control system exists in this genus (Gupta and Fedak, 1985). The system was hypothesized to be polygenic and was reported to be more efficient in tetraploids than in hexaploids, permitting some intergenomic pairing in the latter. Based on the study of meiosis in polyhaploids, the pairing control was inferred to be hemizygous ineffective. It was further concluded that the pairing of homoeologous chromosomes in the wild polyploid *Hordeum* species is generally inhibited by *Hordeum vulgare* and variably enhanced by genomes of *Secale* species.

#### Taxonomy of the tribe Triticeae

Professor Gupta also had a subsidiary interest in taxonomy, which is apparent from his first report of common grasses of Gorakhpur. Later, during his visits to Agriculture Canada in the 1980s, Professor Gupta had a chance to meet and collaborate with Professor Bernard R Baum (a world‐renowned taxonomist) and re‐examined some of the issues, which plagued the taxonomy of the tribe Triticeae. For instance, in association with Professor BR Baum, he criticized the merger of the genus *Aegilops* in *Triticum*, which was based on the argument that *Aegilops* with two of its species contributing to hexaploid wheat can not be recognized as a separate genus. This merger was accepted by the majority of cytogeneticists in North America, and revised names for *Aegilops* within the genus *Triticum* were continuously used for more than two decades. This practice stopped with the publication of criticism by Gupta and Baum (1986), so that the genus *Aegilops* once again regained its position as a valid genus. Similarly, the ‘genomic system of classification’ earlier proposed by Askel Love and DR Dewey was criticized by Professor Bernard Baum jointly with Professor Gupta, since the system was based on an untenable assumption that any two taxa with different genomic constitutions can not be placed within the same genus; this led to unnecessary creation of 19 genera from *Triticum*–*Aegilops* complex. Another taxonomic aspect, which attracted the attention of Professor Gupta was frequent changes in the names of genera within the tribe Triticeae, which he thought was undesirable. They also worked on the taxonomy of the newly established man‐made crop triticale, which could not be given a valid binary name till today and is still treated as a nothogenus, *X Triticosecale*.

### Mutation breeding

In the first half of the 20th century, spontaneous mutations were considered to be an important source for generating novel genetic variation for crop improvement programs. Consequently, major efforts were made to induce mutations in all major crops leading to the release of >3200 cultivars in different crops (FAO/IAEA Mutant Varieties Database). Keeping this in view, Professor Gupta developed a new teaching course on ‘Mutation Research’ prescribed for M. Phil. students, and initiated research in the area of induced mutations. As a result, several Ph.D. students worked on induced mutagenesis in cereals (including wheat and triticale), millets (foxtail millet) (Gupta and Yashvir, 1976) and pulse crops (mungbean). These studies largely included induced variability for a number of simple qualitative traits. In one study involving mutagenic treatments, changes in the magnitude of gene effects and combining ability for quantitative traits like yield and related traits in wheat due to induced genetic variation were examined in F_1_ and F_2_ populations derived from a set of diallel crosses involving treated and untreated material.

The work on induced mutations in mungbean (*Vigna radiata* L. Wilczek) deserves special mention since not only several interesting mutants were obtained in this crop, a mutant cultivar ‘MUM‐2’ was also later approved for commercial cultivation in Northwestern plains in India. The mutant cultivar was approved under All India Coordinated Pulses Improvement Project of ICAR in its meeting held in 1992. This new variety was early and synchronous, high yielding (76.5% higher over K‐851 and 27.9% higher over T‐44, one of the check varieties), and moderately resistant to yellow mosaic virus.

A more recent study on induced mutations involved the use of molecular markers for mapping of induced mutations on individual wheat chromosomes. The novel mutants generated in this study involved the following traits: (i) axillary branching, (ii) early leaf senescence, (iii) reduced number of nodes and (iv) reduced plant height. Such mutants are a good resource for future functional genomics research.

### Studies involving genomic DNA and molecular probes

Starting in mid‐1970s, with the work on nuclear DNA contents, and during the 1980s with the use of molecular probes (RFLPs, etc.) and molecular markers (mainly SSRs), significant contributions were made, which will be described under several subheads.

#### Nuclear DNA content and repetitive DNA

As a British Commonwealth Academic Staff Fellow, Professor Gupta also worked for about 10 months with Professor Hugh Rees, FRS at the University College of Wales, Aberystwyth, UK. During this period Professor Gupta worked out nuclear DNA contents in two closely related species of *Lolium* (*Lolium rigidum* and *Lolium temulentum*), which differed in DNA content by ~30%. By working out DNA contents in F_1_ hybrids and segregating the F_2_ population derived from these two species, he established that the repetitive DNA provided no survival benefit to any plant (Gupta and Rees, 1975). He also worked out for the first time nuclear DNA content in 13 species of the genus *Crotalaria* (Gupta, 1976, 1976).

#### Identification of 1BL‐1RS translocation in Indian wheats: Molecular cytogenetics involving FISH

Molecular cytogenetics involving Fluorescence *In‐situ* Hybridization on mitotic chromosomes was also utilized for a study of the extent of the occurrence of 1BL.1RS translocation in Indian wheats, since 1RS of rye carries genes for resistance to leaf rust, stem rust, stripe rust and powdery mildew, insects and also abiotic tolerance. Besides these resistance/tolerance genes, 1RS has genetic factors for wide adaptation contributing towards higher grain yield. For the purpose of determining the proportion of wheat cultivars with 1BL.1RS translocation, 17 bread wheat genotypes, including Chinese Spring, were screened to identify T1BL.1RS. In eight of these genotypes, the translocation 1BL.1RS was identified using genomic *in situ* hybridization involving total rye genomic DNA as a probe, which allowed identification of a rye chromosome arm carrying a relatively small satellite that is characteristic of 1RS (Kumar *et al*., 2003).

#### Ribosomal DNA polymorphism within the genus Hordeum

During 1986–1988, Professor Gupta worked in close association with Professors Roger Wheatcroft and George Fedak (both at Agriculture Canada, Ottawa) and examined ribosomal DNA polymorphism in 61 accessions belonging to 25 *Hordeum* species. This was later followed by studies in collaboration with Professor E. Nevo of Israel to study ribosomal DNA polymorphism in wild barley (*Hordeum spontaneum*) collections from Israel to study the adaptive role of rDNA polymorphism to climatic variables.

#### Rye repeat sequence in Hordeum and Avena species

During the 1980s, a 120 bp repeat sequence (clone named pSC119) was identified as a repeat sequence that was dispersed throughout the genomes of several species of the tribe Triticeae. This sequence was used as a probe in Southern blots for a search of homology in 61 accessions belonging to 25 *Hordeum* (Gupta *et al*., 1989). Interestingly, except *H*. *vulgare* (cultivated barley) and its related species, *Hordeum agriocrithon* and *H. spontaneum*, homology was observed in all other species. On the whole, DNA hybridization with pSC119 generally gave patterns consistent with the taxonomy of *Hordeum* species, except that *Hordeum bulbosum* and *H. vulgare* were not found to be closely related, since the repeat sequence was missing in *H. vulgare* and *H. spontaeum*, but not in *H. bulbosum*. The authors inferred that this sequence has either been lost or has diverged to such an extent that it is no longer homologous in cultivated and wild barley (Gupta *et al*., 1989). Later, during a 6 months visit to the University of Ottawa, Canada in 1988, Professor Gupta also worked with Professor Illimar Altosaar and developed molecular probes, specific for the A and C genomes of hexaploid *Avena* species.

### Development and use of SSR markers

During the first 20 years (1995–2015) of the establishment of Wheat Biotechnology Laboratory (now MBL), with the help of a dedicated team of workers (including all the three authors of this article), Professor Gupta worked hard for developing SSR markers, preparing genetic and physical maps of these SSRs, and the use of these markers in studying the genetics of a number of quantitative traits in wheat and jute. This work will be briefly described in this section.

#### Development of SSRs in wheat

During the mid‐1990s, while initiating work on the development and use of molecular markers, Professor Gupta was well ahead of time relative to other Indian counterparts. During this period, he joined (as the only member from India) the then recently established Wheat Microsatellite Consortium (WMC) by Professor Peter Isaac from France with an aim to develop and use microsatellite markers for wheat improvement, using a microsatellite enriched genomic library, developed by Professor Keith Edwards from Bristol, UK. During 5 years (1995–2000), WMC developed 500 SSRs, of which at least 50 SSRs were contributed by Professor Gupta and his group at Meerut. Professor Rajeev Varshney also worked on this project for his Ph.D. thesis under the leadership of Professor Gupta. Since these SSR markers were known to be locus‐specific and PCR based, they became markers of choice for developing molecular maps and for molecular breeding involving marker‐assisted selection (MAS). A review article based on this work and future perspectives published in Euphytica in 2000 has been read and cited (>1000) extensively (Gupta and Varshney, 2000).

During 2004–2006, the wheat EST database was also utilized for the development of EST‐SSRs and EST‐SNPs leading to the development of a good collection of at least ~2000 SSRs (including both genomic SSRs and EST‐SSRs). This led to the publication of a series of papers including three important reviews, two on the use of microsatellites in wheat breeding and the third on SSRs from the expressed region of the genome.

#### Development of SSRs in jute

Jute is the second most important fibre crop followed by cotton in the world. The research group led by Professor Gupta initiated work eventually leading to the large‐scale development of SSR markers (~2500 SSRs) in this important fibre crop.

#### Genetic and physical mapping of SSRs in wheat

With the availability of a good collection of genomic SSRs and EST‐SSRs, Professor Gupta, as a leader of the International Wheat Microsatellite Mapping Network, with the help of 16 other scientists across the globe, developed a genetic map for 66 microsatellites (Gupta *et al*., 2002); this map was developed in parallel with other similar SSR genetic maps developed elsewhere. Physical maps involving at least >2000 SSRs, including EST‐SSRs, were also developed.

### QTL mapping in wheat and jute

Professor Gupta and his team were the first in India to utilize interval mapping (IM) of QTLs for several wheat quality traits {grain protein content (GPC), pre‐harvest sprouting tolerance (PHST) and grain weight (GW)}. This work was facilitated by the availability of a number of biparental mapping populations (RILs) developed by Professor H. S. Dhaliwal, Dr. Harjit Singh and their team at Punjab Agricultural University (PAU), Ludhiana. This facilitated identification of molecular markers associated with GPC, initially using single marker analysis involving bulk segregant analysis (BSA) (Prasad *et al*., 1999) and later using linkage‐based interval mapping (Prasad *et al*., 2003).

For QTL IM research, initially one of the major limitations was the development of framework linkage maps, which was demanding, time‐consuming and cost‐ineffective (particularly in India). Keeping this in view, Professor Gupta and his team also utilized the biparental RIL population earlier developed by the International Triticeae Mapping Initiative and utilized for chromosome mapping. As a result, a linkage map for this population was already available and could be conveniently utilized for QTL IM also, thus overcoming the problem of preparing the linkage map, which was a pre‐requisite for QTL IM.

In jute also, after the development of a large repertoire of SSR markers and construction of framework linkage map, QTLs IM and genome wide‐association studies (GWAS) was conducted that led to the identification of QTLs and MTAs for fibre yield and fibre quality traits for the first time (Das *et al*., 2012). This work was extended through collaboration with scientists at ICAR‐CRIJAF, Barrackpore, West Bengal.

#### Chromosome mapping of QTLs in wheat

Initially when markers were utilized for single marker analysis involving BSA, the chromosomal localization of associated markers was achieved using nullisomic‐tetrasomic lines, ditelocentrics and deletion lines of wheat (Varshney *et al*., 2000a). This was successfully achieved in at least three studies, the first involving GPC (Prasad *et al*., 1999), PHST (Roy *et al*., 1999) and GW (Varshney *et al*., 2000b). The approach was also later used for physical mapping of SSRs, as described earlier in this review.

Although the era of development of whole‐genome linkage maps and QTL IM started during the early 1990s, no expertise for conducting IM utilizing software like QTL Cartographer was available in the country. This problem was initially overcome by a visit of Professor Gupta to North Carolina State University (NCSU) in Raleigh, NC, USA where his son was working at the famous Research Triangle Park. This allowed him in 2000 to participate there in the ‘Summer Institute in Statistical Genetics’, which was an annual feature at NCSU. The development of the relationship with Professor Z B Zeng of NCSU also allowed Professor Gupta to visit NCSU intermittently during 2000–2012. This facilitated Professor Gupta and his coworkers to initiate work in the field of IM and association mapping leading to the publication of a series of papers. Later, this also prompted Professor Gupta to write a book on ‘*Quantitative Genetics*’ (published in 2020, by Rastogi Publications, Meerut, India) for graduate students in India.

#### Two‐locus QTL analysis

Regular visits to NCSU allowed Professor Gupta to transfer the technology of QTL IM involving the use of QTL Cartographer to Meerut. However, initial studies involved identification of only main effect QTLs, but no Q x Q interactions could be examined; however, this became possible later with the availability of QTLMapper/QTLNetwork developed by Professor Jun Zhu and his team in China. The technology was immediately picked up by Professor Gupta and his students to conduct a two‐locus QTL analysis. Association with Professor Jun Zhu also prompted Professor Gupta to visit China and attend the ‘3rd International Conference on Quantitative Genetics (ICQG)’, organized by Professor Jun Zhu at Zhejiang University in Hangzhou in 2007. Following his visit to China, Professor Gupta also organized an ‘Indo‐Chinese Bilateral Workshop on Plant Genomics and Quantitative Genetics’ at Meerut during 14–16 February, 2009, which was attended by several eminent scientists from China (including Professors Qifa Zheng, Jun Zhu, and Bin Han) and by the renowned expert in statistical genetics, Professor ZB Zeng from NCSU, USA.

### Genome wide‐association studies

Professor Gupta also believed that if one wants to understand and conduct research in a new area, he/she should plan to write a review on that subject to learn about the available research already done and understand the basics about the subject. Following this practice, Professor Gupta initially wrote a review on linkage disequilibrium and association mapping (Gupta *et al*., 2005, with >800 citations), and later wrote two more reviews to cover the literature on this fast‐developing subject of association mapping (Gupta *et al*., 2014, 2019). He realized the importance of the work on association mapping because, as widely known now, only a very limited genetic variation is sampled during IM involving the use of biparental mapping populations. In contrast, association mapping makes use of a large number of accessions from the natural populations, which include almost the entire genetic variability of the crop and are the product of thousands of recombinations, thus improving the resolution. Thus in parallel with work on IM, the dedicated team in the laboratory of Professor Gupta conducted GWAS for a variety of traits in wheat and jute.

### Development of a WheatQTLdb

More recently, Professor Gupta realized that a very large number of QTLs for almost all kinds of traits in wheat have become available, but no database for wheat QTLs was available. To fill this gap, his group developed a comprehensive database of all the QTLs identified in wheat (Singh *et al*., 2021); the database is already being utilized by several groups around the world including Professor Rudi Appels and his group in Australia.

### MetaQTL analysis

Recognizing the availability of a large number of QTLs for each individual trait in a crop like wheat, and also the current failure to utilize these QTLs for MAS, Professor Gupta initiated work involving metaQTL analysis in wheat (also including identification of ortho‐metaQTLs and candidate genes). This work already led to a series of publications and the activity still continues with several papers in the pipeline. Hopefully, the metaQTLs and the ortho‐metaQTLs identified through these studies will provide more robust markers to be used for MAS.

#### Molecular breeding involving improvement of grain quality and disease resistance in wheat

The QTLs/genes and the associated markers once identified and validated, are deployed into molecular breeding programs aimed at the development of next‐generation crop varieties with enhanced agronomic performance. Professor Gupta took the lead and helped DBT, Govt. of India (as Chairperson for a Task Force constituted for evaluation of proposals for funding in the area of MAS). The Task Force sanctioned funds for several multi‐institutional projects involving collaboration between participating institutes in India. His own group was also actively involved in MAS/pyramiding of different QTLs/genes in wheat for the following traits in wheat: (i) GPC; (ii) PHST; (iii) GW; (iv) water use efficiency (drought tolerance) and (v) disease resistance. The pre‐bred material, including near‐isogenic lines for GW developed through these studies will serve as an important plant genetic resource for future wheat breeding programs.

## A successful mentor

**Figure 3:**
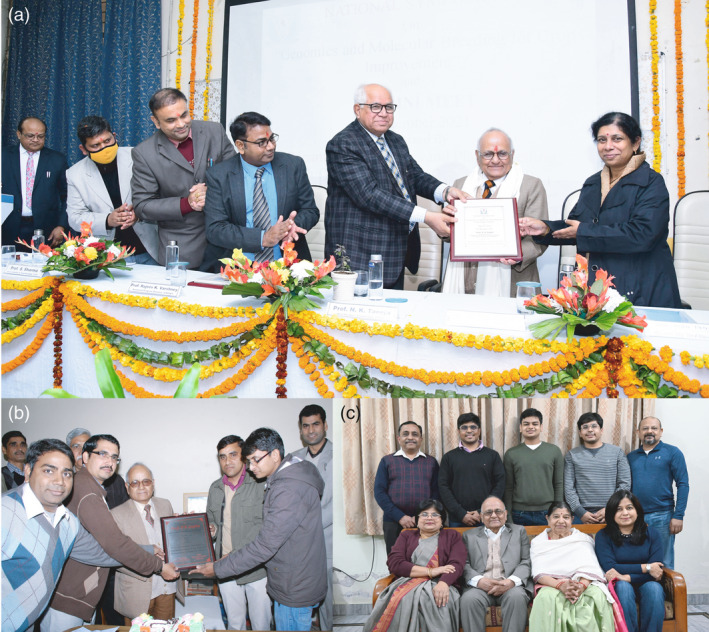
Professor PK Gupta with students/scientists and his family members during different events. (a) Professor Gupta was felicitated by Vice‐Chancellor, Ch. Charan Singh University, Meerut and faculty members of Department of Genetics and Plant Breeding and Prof. Rajeev K. Varshney on his 85th birthday celebrated in December 2021. (b) Professor Gupta with his students during the celebration of his 75th birthday in 2011. (c) Professor PK Gupta with his family (Box 2). The picture shows from left to right (first row), Ritu Gupta (daughter), Prof. PK Gupta, Sudha Gupta (wife) and Gunjan Gupta (daughter‐in‐law). In the second row from left to right, Mukul Gupta (son‐in‐law), Naman Gupta (grand son), Abhay Gupta (grand son), Manas Gupta (grand son) and Ankur Gupta (son).

In India, Professor Gupta is widely known as one of the most successful mentors in the area of plant genetics and genomics. He mentored >80 students for Ph.D. degrees with diverse research topics like cytogenetics, mutation breeding, quantitative genetics and various disciplines of plant genomics. A number of students after graduating from other universities worked with him and now occupying important positions, not only in India but also in United States and Canada. The contributions of Professor Gupta in training human resource is widely recognized as a service to the nation. The famous quote that ‘Growing old is mandatory but growing up is optional’ perfectly suits Professor Gupta. He enjoys working even at the age of 85 years because of his love and care for his students and devotion to science. He is really an example of a teacher who enjoys nurturing a new generation of students. The love and affection shown by Professor Gupta towards his students were also reciprocated equally by the students. That’s the reason that students celebrated his 75th birthday in 2011 (Figure 3), 80th birthday in 2016 and 85th birthday in 2021 (Figure 3), the latter two associated with the organization of a national symposium on ‘Genomics and Molecular Breeding’ by the department, where a large number of his students as well as alumni of the department and scientists from India and abroad participated.

## A voracious reader/writer

Professor Gupta has a thirst for knowledge (as described by Professor George Fedak in a message on his 80th birthday) associated with a strong urge for writing on the emerging subject areas. His writing work includes textbooks, review articles and original research papers for the student and teacher community. Starting in 1974 with his first book entitled ‘*Cytology, Genetics and Evolution*’ (also initially subsidized by National Book Trust of India), Professor Gupta has written and edited more than two dozen such books; some of these books, translated in Hindi, also won awards from ICAR and DBT. The subject areas on which he has written books include genetics, cytogenetics, biotechnology, cell biology, molecular biology, genomics and quantitative genetics (Table 2). The textbooks on genetics, biotechnology and genomics and plant biotechnology are very popular amongst the students in India and abroad. The book ‘Cytogenetics: An Advanced Text Book’ written by him is also used for teaching in several countries including United Kingdom, United States and Canada as a standard textbook on cytogenetics. This is borne out by several interactions and communications that Professor Gupta had with teachers from United Kingdom (Professor RN Jones from, Aberystwyth, Wales), United States (Professor David Weber, University of Illinois) and Canada (University of Dalhousie, Halifax, Canada).

**Table 2:** Books written/edited by Professor P. K. Gupta

S. No	Title	Year	Publisher
Cytogenetics			
1	A Text Book of Cytology, Genetics and Evolution	1974, 6th edition 2000	Rastogi Publications, Meerut, UP, India
2	Cytogenetics of Crop Plants (Eds. MS Swaminathan, PK Gupta and U Sinha)	1983	Macmillan India Limited
3	Cytogenetics: An Advanced Text Book	1995	Rastogi Publications, Meerut, UP, India
4	Chromosome Engineering in Plants: Genetics, Breeding, Evolution Part A and Part B (edited along with T Tsuchiya)	1991	Elsevier Science Publishers
Genetics			
5	A Text Book of Genetics	1985; 5th edition 2018	Rastogi Publications, Meerut, UP, India
6	Cell Biology and Genetics	1985; 4th edition 2018	
7	Genetics and Cop Improvement’ (edited along with JR Bahl)	1986	
8	Genetics and Biotechnology in Crop Improvement (edited along with SP Singh, HS Balyan, PC Shama, B. Ramesh)	1998	
9	Concepts in Genetics	1999; 3rd edition 2014	
10	Genetics: Classical to Modern	2005	
11	Quantitative Genetics	2020	
Biotechnology/Molecular Biology/Cell Biology			
12	Elements of Biotechnology	1994; 2ndedition 2010	Rastogi Publications, Meerut, UP, India
13	Elements of Plant Biotechnology	1999; 2nd edition 2018	
14	Cell Biology	1999; 3rd edition 2014	
15	Molecular Biology	1999; 3rd edition 2014	
16	Cell and Molecular Biology	1999; 3rd edition 2014	
17	Biotechnology and Genomics	2003	
18	Molecular Biology and Genetic Engineering	2005	
19	Plant Biotechnology	2010	
20	Biotechnology and Immunology	2013	
21	Molecular Biology and Biotechnology	2014	
22	Animal Biotechnology	2017	
23	Biomolecules and Cell Biology	2017	
Genomics			
24	Cereal Genomics (edited along with R.K. Varshney)	2004	Kluwer Scientific Publishers, The Netherlands
25	Cereal Genomics II (edited along with R.K. Varshney)	2013	Springer, Germany

## Awards and honours received

Professor Gupta is a Fellow of all the four national science/agriculture academies of India, including the Indian National Science Academy (FNA), the Indian Academy of Sciences (FASc), the National Academy of Sciences India (FNASc) and the National Academy of Agricultural Sciences (FNAAS). Besides this, he has been a fellow and a member of several academies and scientific societies in the country. Important awards won by him include Vishwavidyalaya Gaurav award by Meerut University (2003), Birbal Sahni Gold Medal of the Indian Botanical Society (2004), ‘Outstanding Researcher’ Award by Society for the Promotion of Plant Science Research, Jaipur National University, Jaipur (2013) and the Association of Biotechnology Led Enterprises (ABLE) Award for ‘Excellence in Agricultural Research’ (2013) .

Box 2 **Family life: A wonderful husband and a caring father**

Professor Gupta has been fortunate to have a life partner (Sudha; married in 1964), who helped him and continues to do so, both in his professional activities and household; therefore, to some extent, he owes his professional success to his wife, who helped him in a variety of ways including all kinds of typing work till recently, when he switched to use of a computer for all his writing work. She also joined his lab as a technician for a brief period (1980‐1988) and helped in cytological work including study of meiosis in a difficult material like lentil. His elder sibling is a daughter, Dr. Ritu Gupta (MBBS), married to Mukul Gupta, MD (both practicing medicine), and the younger sibling, Ankur Gupta (B. Tech, Computer Science), married to Gunjan Gupta (MBA, NCSU, USA), both currently working in foreign Banks (Deutsche Bank and Barclays Bank). He has three grandsons, Abhay (born to Ankur and Gunjan in 2004, and currently studying at Mahindra United World College, near Pune) and Naman, B. Tech, BITS Goa; MS CMU, USA and Manas, MS, Applied Mathematics, IIT Roorkee (both born to Ritu and Mukul in 1994 and 1998, respectively), the elder working as a Software Engineer (Robotics Simulation) with Johnson and Johnson in California USA and the younger working as Quantitative Research Analyst, with JP Morgan Chase & Co., Mumbai, India. Thus, Professor Gupta completed 57 years of happy married life (Figure 3c). According to him, he also has an extended family, which includes dozens of his formal and informal students, whom he loves.

## Conflict of interest

The authors declare no competing interests.

## Author contributions

Rajeev K. Varshney conceptualized the idea of developing the biography and coordinated the development and finalization of the article. Pawan L. Kulwal took lead to synthesize different sections and Reyazul Rouf Mir contributed to specific sections and collaborated with Rajeev K. Varshney and Pawan L. Kulwal to finalize the article.
